# “Steak Dry Aging”: An Innovative Approach to Producing High‐Yield, High‐Quality Dry‐Aged Beef

**DOI:** 10.1111/1750-3841.71029

**Published:** 2026-03-28

**Authors:** Jean Carlos dos Santos, Angélica Sousa Guimarães, Lorrany Ramos do Carmo, Márcia Cristina Teixeira da Silveira, Alcinéia de Lemos Souza Ramos, Leandro Sâmia Lopes, Eduardo Mendes Ramos

**Affiliations:** ^1^ Department of Food Science, School of Agricultural Sciences of Lavras Federal University of Lavras Lavras Brazil; ^2^ Department of Water, Soil and Environmental Sustainability Embrapa Maize and Sorghum Sete Lagoas Brazil; ^3^ Department of Animal Science, Faculty of Animal Science and Veterinary Medicine Federal University of Lavras Lavras Brazil

**Keywords:** beef quality, free amino acids, saleable yield, Stepwise dry/wet aging

## Abstract

This study evaluated a novel steak dry‐aging (StDA) protocol in which portioned beef steaks were dry‐aged until reaching an average weight loss of 15% and subsequently wet‐aged to achieve a total aging time of either 14 or 28 days. The new protocol was compared with conventional dry aging (DA) for 28 days and unaged controls. Under the experimental conditions, StDA increased saleable yield (up to 35% higher than DA) and reduced trimming steps during processing. Physicochemical composition, water activity, total free amino acids, and microbiological quality (< 3.5 log CFU/g) were similar between DA and StDA samples. StDA aged for 28 days had a free amino acid profile comparable to DA, but with higher glutamic acid content (2.30 vs. 0.80 mg/g protein), associated with umami flavor, and lower taurine content (0.59 vs. 1.13 mg/g protein). DA samples had lower shear force (27.45 vs. 35.54 N) and a lighter (L* = 43.39 vs. 38.77) and more intensely (C* = 26.66 vs. 21.31) red color. Overall, 28‐day StDA combined high yield, preservation of sensory‐related attributes, and process efficiency, representing a viable and scalable alternative for producing high‐quality dry‐aged beef.

**Practical Applications:**

The data from this study demonstrate that the “Steak Dry Aging” method offers a practical way for processors to produce dry‐aged beef with higher yield, less waste, and shorter production times. Because it avoids heavy trimming and allows part of the aging to occur during distribution, it can be easily adopted by small and medium facilities. This makes premium dry‐aged beef more affordable, offering a novel and economically viable alternative for the meat sector.

## 1 Introduction

Ensuring the supply of high‐quality beef is essential to meet consumer demands and strengthen the meat industry. Aging is a well‐established practice that enhances attributes such as tenderness, juiciness, aroma, and flavor (Guimarães et al. 2025). In particular, dry aging intensifies sensory characteristics by concentrating volatile compounds and activating endogenous enzymes under controlled conditions of temperature, humidity, and airflow (Álvarez et al. 2021; Ramanathan et al. 2020). Although still relatively new in the Brazilian domestic market and lacking specific regulations for its production and commercialization, the dry‐aged beef market has been growing in Brazil, especially in large urban centers, driven by consumer demand for premium products with distinctive sensory profiles. This growth, albeit limited, has fostered the emergence of small specialized businesses such as gourmet butcher shops, restaurants, boutiques, and emporiums that operate on a small scale (Rezende‐de‐Souza et al. 2021). Moreover, dry‐aging presents expanding business opportunities globally, as consumers in different countries increasingly seek premium and gourmet products with unique sensory qualities, highlighting its commercial potential beyond the Brazilian context. According to Ribeiro et al. (2025), the global dry‐aged beef market is a high‐value, specialized segment predominantly concentrated in North America, mainly the United States, which accounts for over 40% of global consumption. Europe follows with approximately 30% of the global volume, driven by growing interest in specialty butcher shops in Spain, Italy, Germany, the United Kingdom, and France. Asia has also shown a significant increase in demand, particularly in China, Korea, and Japan, where dry‐aged Wagyu commands premium prices.

Recent studies (Berger et al. 2018; Guimarães et al. 2025) have shown that dry aging improves the palatability of grass‐fed beef with low marbling, suggesting its potential to add value to Zebu beef commonly marketed in tropical countries such as Brazil. However, the adoption of high‐cost methods such as dry aging depends on consumer acceptance and the perceived value of sensory improvements. Evidence indicates that consumers are willing to pay a premium for beef with more intense flavor and differentiated texture, particularly in gourmet and fine dining niches, reinforcing the commercial potential of the dry aging process (Ortez et al. 2022; Setyabrata et al. 2022). Nonetheless, large‐scale adoption is constrained by economic and operational barriers, including the need for specialized infrastructure, strict environmental control, extended processing times, skilled labor, and high processing losses due to meat's water evaporation during aging and the removal of the dried crust surface that forms. Combined evaporation and trimming of the dried crust can reduce saleable yield to 60%–65% of the initial weight, undermining commercial viability (Álvarez et al. 2021; Dashdorj et al. 2016). For small and medium‐sized meat companies, limited storage capacity and reluctance to accept high yield losses represent major obstacles to adopting dry aging (De Faria Vilella et al. 2019; Haddad et al. 2022).

In recent years, new strategies have been developed to minimize these losses, such as packaging with high water vapor permeability (in‐bag dry aging) (Ahnström et al. 2006; Dikeman et al. 2013; Li et al. 2014; Setyabrata et al. 2022) and combined approaches in which dry‐ and wet‐aging are sequentially applied (stepwise dry/wet aging) (Kim et al. 2017; De Faria Vilella et al. 2019; Correa et al. 2025). However, despite these innovations, their impact on reducing losses—particularly trimming losses—remains limited, and the process continues to be costly. Furthermore, because aging improves flavor and tenderness through enzymatic activity and oxidation reactions over time (Terjung et al. 2021), simply shortening the aging period does not ensure the development of the characteristic dry‐aged profile. While moisture loss through evaporation is essential for flavor concentration and reaction development, losses due to crust formation may not be indispensable.

Dry aging is traditionally applied to whole carcasses, primal or subprimal cuts, or large sections, and limited information is available on the aging of small portions, such as steaks. Adapting this process to individual steaks requires careful consideration of microbiological safety, as increased surface area and handling may favor contamination in the absence of strict hygienic and environmental controls. Therefore, this study evaluated whether a stepwise dry/wet aging process applied to individual steaks, rather than to large cuts, could eliminate yield losses associated with surface trimming during dry aging, thereby optimizing the process and expanding its applicability. We hypothesized that individual steak drying would promote rapid evaporative water loss, concentrating solutes and substrates required for aging‐related biochemical reactions while minimizing surface crust formation. Subsequently, a wet aging phase would enable rehydration of the thin crust formed, while allowing continued enzymatic activity and oxidative reactions. Accordingly, this study aimed to develop a novel dry‐aging strategy for steaks, termed “steak dry aging” (StDA), to eliminate trimming losses and improve process yield, while advancing technical knowledge through the evaluation of physicochemical, technological, microbiological, and amino acid profile parameters.

## 2 Materials and Methods

### 2.1 Sample Collection and Aging Treatments

**FIGURE 1:**
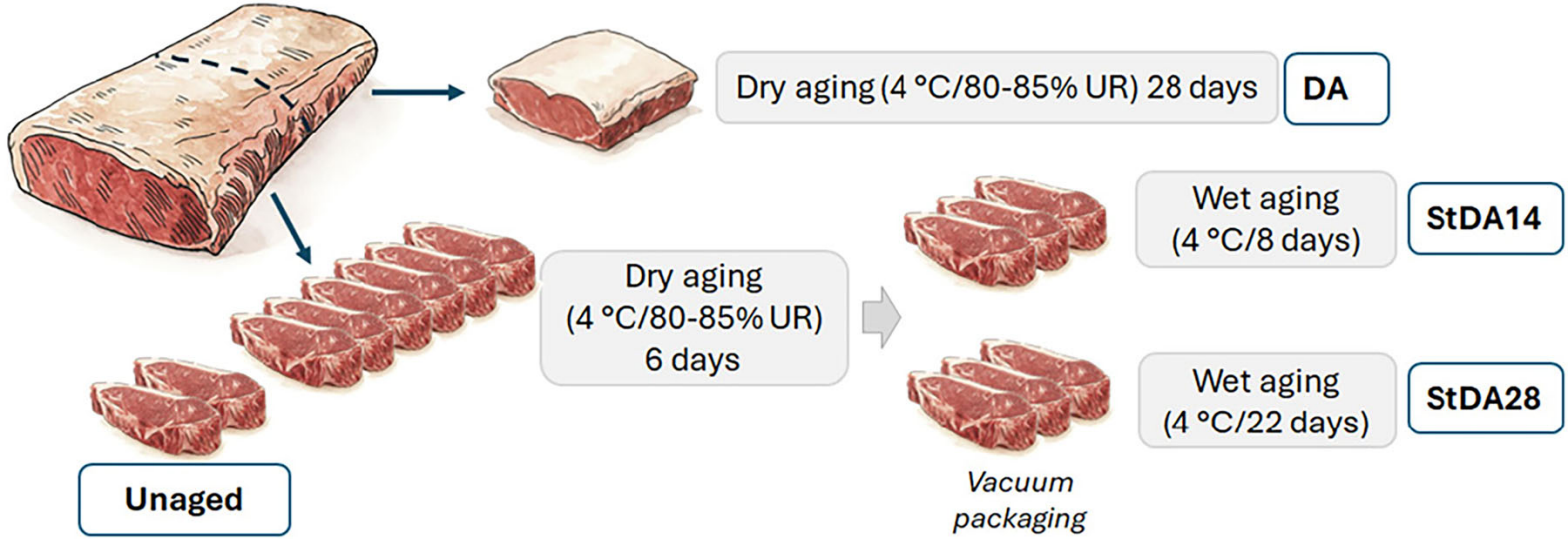
Schematic representation of treatment sampling.

Ten boneless striploin cuts (M. *Longissimus lumborum*) were collected from 10 heifers (18 months old; Nellore and Nellore×Angus crossbreeds), raised on pasture and finished in feedlots. Sampling was performed 72 h postmortem at a commercial slaughterhouse under Federal Inspection Service (SIF) supervision in Sete Lagoas, MG, Brazil. The striploins were packaged in plastic film (non‐vacuum) and transported in ice‐cooled containers to the Laboratory of Meat Science and Technology (LabCarnes) at UFLA, where they were properly identified. Each striploin was divided into two portions (Figure 1): one ∼15 cm portion from the caudal region, destined for the dry aging (DA) treatment; and another ∼25 cm portion, from which eight steaks of approximately 3.0 cm thickness were obtained. Six steaks were randomly assigned to the steak dry aging (StDA) treatment, and the remaining two were allocated to the control group (Unaged).

**FIGURE 2:**
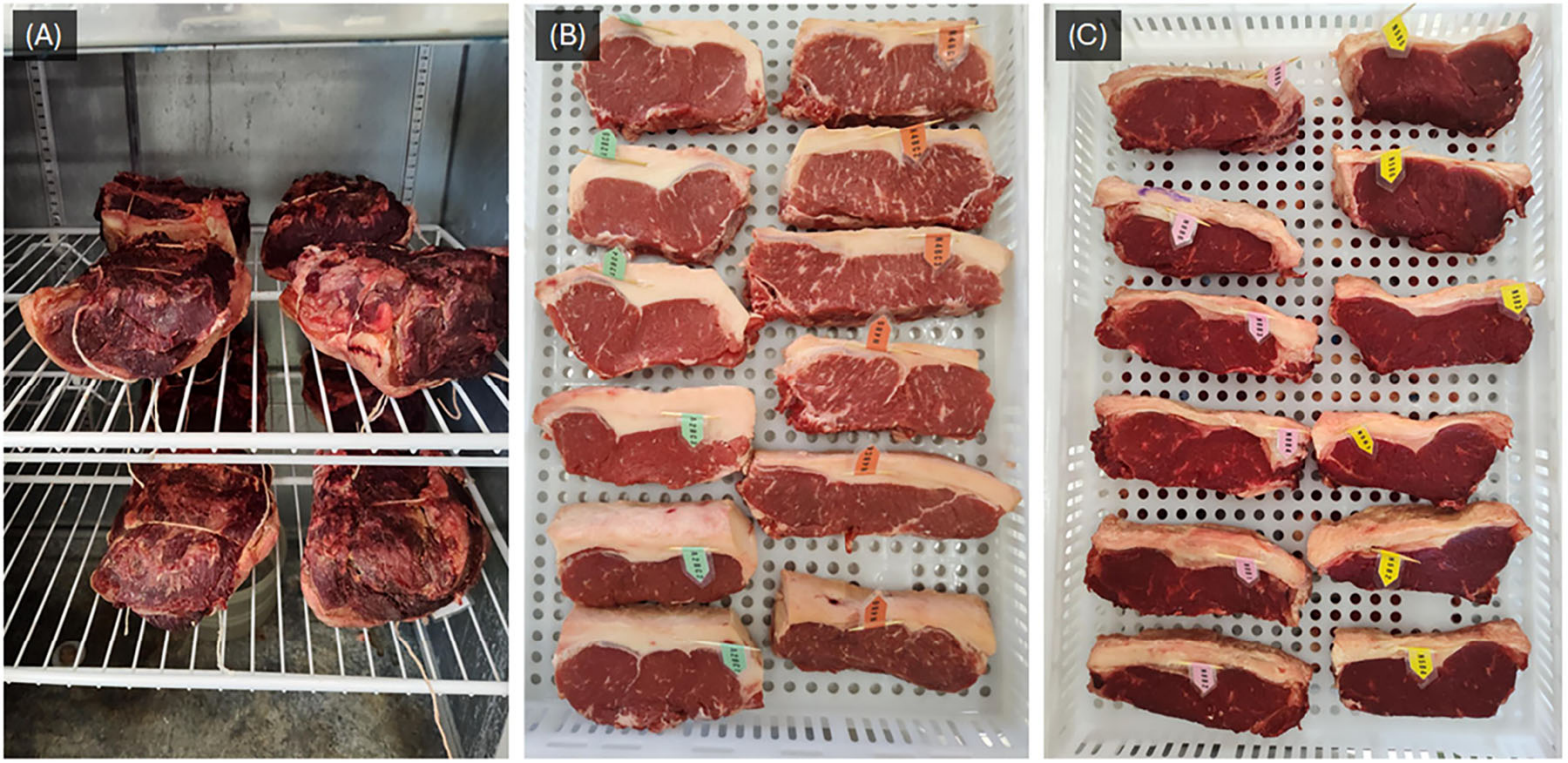
Photos of (A) dry aging (DA) chamber and striploin steaks (B) before and (C) after six days of drying in the StDA treatment.

The portions designated for the DA treatment were weighed, labeled, and stored in a four‐door commercial refrigerator (model GREP‐4P; Gelopar, Chapada Araucária, PR, Brazil) with low air circulation (<0.2 m/s) for 28 days (Figure 2A). During aging, chamber temperature (4.8°C ± 0.6°C) and relative humidity (82.4% ± 7.6%) were monitored using a digital thermo‐hygrometer datalogger (AK174; AKSO Produtos Eletrônicos, São Leopoldo, RS, Brazil). The samples were weighed weekly, and their shelf positions rotated. After aging, the dried surface (external crust) was trimmed and weighed to calculate losses, and the remaining meat was cut into ∼2.5 cm thick steaks for subsequent analyses.

The steaks (six for each striploin) assigned to the StDA treatment were individually weighed and labeled (Figure 2B), then stored in a climatic chamber (BOD model EL202; EletroLab, São Paulo, SP, Brazil) with low air circulation (<0.2 m/s), at an average temperature of 4.2°C ± 0.8°C and relative humidity of 84.8% ± 6.9%, monitored by a digital thermo‐hygrometer datalogger (HT550; Instrutherm, São Paulo, SP, Brazil). The steaks were weighed daily and kept in the chamber until reaching an average weight loss of 15% (by six days; Figure 2C). At this point, they were individually vacuum packaged (BS420; R. Baião, Ubá, MG, Brazil) and returned to the same chamber (at 4°C) for wet aging, with three steaks randomly assigned to a total aging of 14 days (StDA14) and three for a total of 28 days (StDA28), including both dry and wet aging periods. The target weight loss of 15% during the StDA drying phase was determined in preliminary tests, in which an adequate balance between steak shrinkage during drying and the rehydration capacity of the crust formed after vacuum storage was visually assessed (Figure S1). A minimal shrinkage (including the difference between the muscle surface and subcutaneous fat) and the absence of areas with dry crusts on the surface were primary parameters for choosing the degree of dryness of the steaks in the experiment.

### 2.2 Analyses

#### 2.2.1 Mass Losses

Mass losses during the aging process were determined by weighing the samples before and after each treatment stage, with results expressed as a percentage of the initial weight. Evaporation loss was calculated as the difference between the initial weight (time zero) and the final weight after aging for DA samples, or after the drying stage for StDA samples. Trimming loss was calculated as the difference between the weight before and after removal of the outer crust in the DA portions. Exudation loss (purge) in StDA samples was determined as the difference between the weight before vacuum packaging and after the wet aging period. Finally, total yield was expressed as the percentage ratio of the final weight of the ready‐to‐sell products to the initial sample weight.

#### 2.2.2 Water Retention Capacity

Water retention capacity was assessed by drip loss (DL) and cooking loss (CL) methods. DL was determined according to the EZ‐DripLoss (EZ‐DL) method described by Torres Filho et al. (2017). Using a 2.5 cm diameter cylindrical mold, two samples were obtained from the steak, placed in EZ‐DL tubes (with lids and funnel‐shaped openings), and stored (at 4°C) for 48 h. After storage, the samples were removed, the tubes were weighed with exudate, and the percentage of mass loss was calculated concerning the initial sample weight. For the CL, the steak (without the subcutaneous fat) was cooked on a preheated grill at 200°C (Model SCGE; Croydon; Duque de Caxias, Rio de Janeiro, Brazil) until reaching an internal temperature of 70°C, monitored by a digital thermometer (Chugod‐Smart Wireless BBQ Thermometer, China) inserted in the geometric center. Steaks were weighed before and after resting for 1 h (at room temperature) after cooking to determine CL, expressed in percentage.

#### 2.2.3 pH, Chemical Composition, and Water Activity

The pH was measured by direct penetration at three different points of each sample, avoiding areas containing visible fat or connective tissue, using a portable pH meter (206‐pH2; Testo do Brasil, Campinas, SP, Brazil). The chemical composition (protein, moisture, fat, and ash) and water activity (aw) were determined in lean ground steak samples. Composition was analyzed by near‐infrared spectroscopy (NIR; AOAC method 2007‐04) using a FoodScan device (FOSS Analytical A/S, Hillerød, Denmark), while aw was measured with an Aqualab CX2 device (Decagon Devices Inc., Pullman, WA, USA).

#### 2.2.4 Miofibrilar Fragmentation Index

The degree of myofibrillar fragmentation was assessed using the Myofibrillar Fragmentation Index method (MFI), as described by Aroeira et al. (2020), with minor modifications. Approximately 3 g of finely minced sample was homogenized (Turratec TE 102; TECNAL, Piracicaba, SP, Brazil) in 15 mL of cold (4°C) extraction buffer (20 mM potassium phosphate, 100 mM potassium chloride, 1 mM magnesium chloride, 1 mM sodium azide, 1 mM disodium EDTA; pH 7.0) for 15–30 s. The homogenate was centrifuged (Hettich‐EBA 21; Nova Analítica Importação e Exportação Ltda., São Paulo, Brazil) at 3000×g for 5 min, and the supernatant was discarded. The pellet was resuspended in 10 mL of extraction buffer, vortexed for 10 s, and centrifuged again at 3000 × g for 5 min. The supernatant was discarded, and this extraction procedure was repeated twice. Finally, the pellet was resuspended in 10 mL of extraction buffer, and protein concentration was determined by the Biuret method (Ramos and Gomide 2017). An aliquot of the myofibril suspension was then diluted with extraction buffer to a final protein concentration of 0.5 mg/mL. Absorbance was measured at 540 nm in a spectrophotometer (Genesys 10 UV; ThermoScientific Varian, São Paulo, Brazil) against the extraction buffer blank. The mean absorbance values were multiplied by 200 to adjust the scale to a range of 30–100, defined as the MFI, with higher values indicating greater proteolysis.

#### 2.2.5 Shear Force

Shear force was measured in the cooked samples used for CL analysis (section 2.2) following the Warner–Bratzler square shear force (WBsSF) method described by Silva et al. (2015). After cooking, the samples were kept packaged at 4°C for 12 h prior to WBsSF analysis. Six rectangular sections (1.0 × 1.0 × 2.5 cm) were excised from each sample, cut parallel to the muscle fibers orientation, and sheared perpendicularly at a crosshead speed of 3.33 mm/s using a Warner–Bratzler V‐blade attached to a TA.XTplus texturometer (Stable Micro Systems Ltd., Godalming, Surrey, UK). The maximum force (N) required to completely shear each section was recorded, and the mean value was taken as the WBsSF of the sample.

#### 2.2.6 Myoglobin Redox Forms and CIE Color

The instrumental color of the samples was evaluated using a CM‐700 portable spectrophotometer (Konica Minolta Sensing Inc., Osaka, Japan) equipped with an 8 mm port aperture (MAV), illuminant A (incandescent, tungsten filament, 2857 K), 10° standard observer angle, and operating in both specular components excluded (SCE) and included (SCI) modes. Lightness (L*), redness (a*), yellowness (b*), chroma (C*), hue angle (*h*, °), and spectral reflectance data from 400 to 710 nm (10 nm intervals) were obtained as the average of five readings on the steak surface after 30 min of blooming at room temperature. Based on the reflectance spectrum, myoglobin chemical pigments were quantified following the method of Krzywicki (1979), as described by Bueno et al. (2024). Reflectance (R) values at isosbestic points (525, 473, and 572 nm) were used to calculate the relative proportions of deoxymyoglobin (DMb), metmyoglobin (MMb), and oxymyoglobin (OMb).

The redox forms of myoglobin (OMb, DMb, and MMb) on the beef surface were also estimated using a computer vision system (CVS) with a multilayer neural network algorithm (Meat Mb Redox software, registered at the National Institute of Industrial Property: BR512023 000048‐5) described by Bueno, Silva, et al. (2024). Images were captured in a cuboid photography chamber (equipped with four 3.5 W LED lamps, 2700 K color temperature; Opus, model LP 37097) using a Canon DSLR T3i camera. The images were saved in RAW format and subsequently calibrated with a ColorChecker card to generate a color calibration profile in Adobe Lightroom Classic software.

#### 2.2.7 Microbial Growth

The unaged and aged samples (DA and StDA) were analyzed for total bacterial counts (TBC) following the method of Silva et al. (2017). Approximately 25 g of each sample was aseptically collected, placed in plastic bags containing 225 mL of 0.1% peptone water, and homogenized in a stomacher (Metroterm, Brazil) at 490 strokes/min for 3 min. Serial decimal dilutions were prepared in tubes containing 9 mL of 0.1% peptone water. Colony‐forming units (CFU) were enumerated after incubation at 37°C for 24 h on Plate Count Agar (PCA) and expressed as log CFU/g.

#### 2.2.8 Lipid Oxidation

Lipid oxidation was evaluated by determining thiobarbituric acid reactive substances (TBARS), according to modifications of the method described by Pikul et al. (1989). Approximately 5 g of sample was weighed and homogenized in 15 mL of 3.86% perchloric acid and 1 mL of 0.15% hydroxybutyltoluene (BHT) antioxidant for approximately 30 s. Then, the homogenate was filtered through filter paper, and a 2 mL aliquot of the filtrate was added to 2 mL of 0.02 M thiobarbituric acid (TBA; in 3.86% perchloric acid), vortexed and heated in a water bath at 90°C for 30 min. The samples were cooled in an ice bath for 3 min, and the absorbance reading at 532 (Genesys 10 UV‐Vis Spectrophotometer; Thermo Fisher Scientific Inc., Waltham, EUA) was obtained. TBARS values (mg malondialdehyde—MDA/kg) were determined from an analytical curve with 1,1,3,3‐tetraethoxypropane (TEP).

#### 2.2.9 Free Amino Acids

Samples of raw meat (∼30 g) were cut into pieces, stored at −75°C (CL120‐86 V; Coldlab, Piracicaba, SP, Brazil), and freeze‐dried (L4KR; Edwards, São Paulo, Brazil) for free amino acid (FAA) analysis. FAAs were extracted using a methanol:0.1 M HCl solution (60:30 v/v) and quantified by high‐performance liquid chromatography (HPLC) following the procedure of Hagen et al. (1989). Analyses were performed on Shimadzu HPLC equipment (Shimadzu Corporation, Tokyo, Japan) equipped with a C18 column (100 Å, 5 µm, 250 × 4.6 mm; Phenomenex, Torrance, CA) and UV detection at 254 nm after pre‐column derivatization with phenylisothiocyanate (PITC). Alpha‐amino butyric acid was used as an internal standard, and sodium acetate trihydrate and acetonitrile served as the mobile phase. Under these conditions, asparagine was hydrolyzed to aspartic acid and glutamine to glutamic acid; therefore, reported values represent the combined amounts of these respective components. The content of each AA was expressed as mg/g of protein, determined by the Kjeldahl method (AOAC 928.8) (AOAC 2012).

### 2.3 Statistical Analysis

The experiment was conducted using a randomized block design (RBD), with each striploin (from each animal) considered a block (n = 10). Main effects were analyzed by analysis of variance (ANOVA), with the type of aging (unaged, DA, StDA14, and StDA28) as the source of variation, and post‐hoc comparisons of means were performed using Tukey's test at a 5% significance. Statistical analyses were carried out using Statistica 8.0 software (StatSoft Inc., Tulsa, OK, USA). For mass losses during the drying stage, sigmoidal trendlines were fit using SigmaPlot 12.0 software (Grafiti LLC; Palo Alo, CA, EUA).

## 3 Results and Discussion

### 3.1 Mass Losses and Retail Yield

**FIGURE 3:**
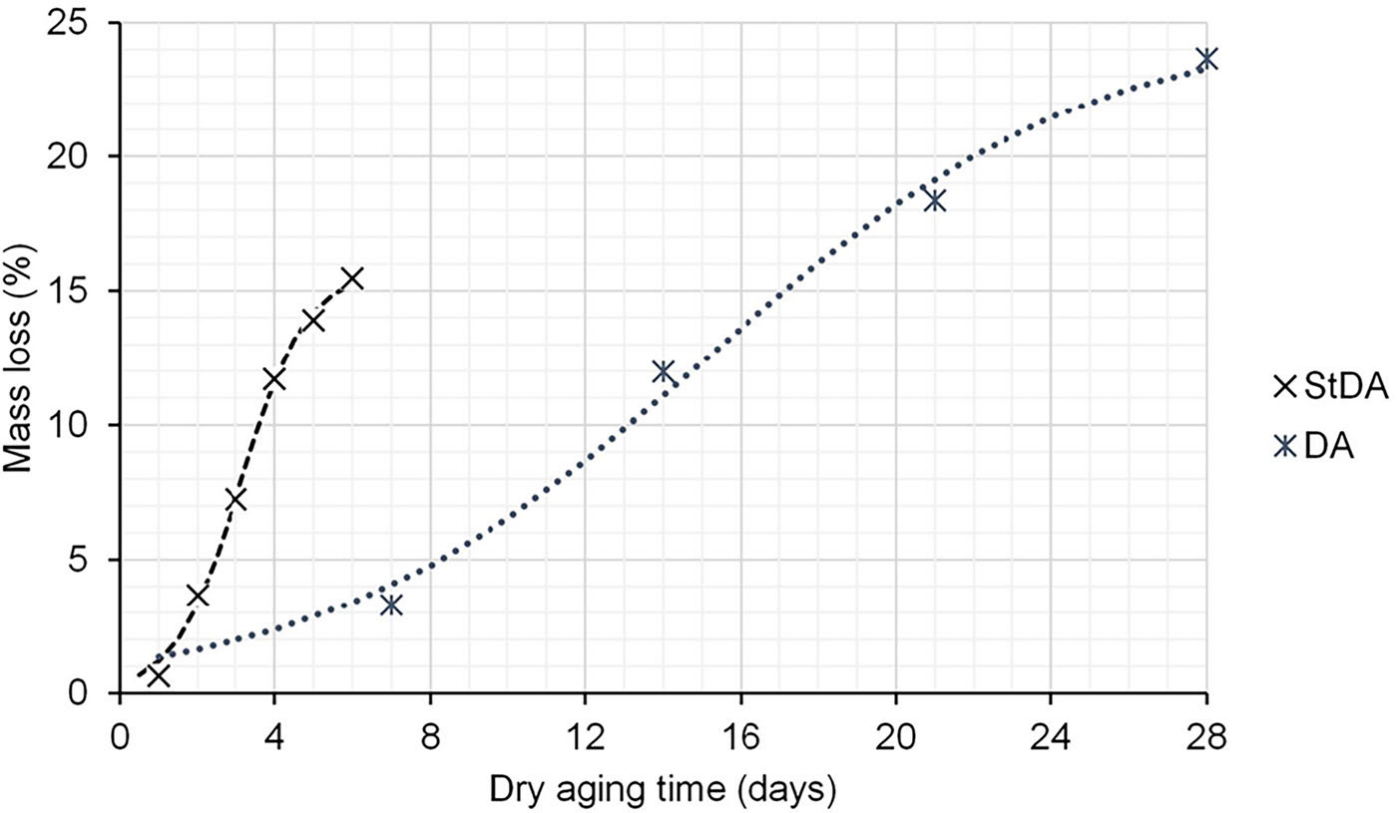
Average mass loss (ML) of beef striploin (M. *L. lumborum*) steaks (StDA; before vacuum‐packaging and wet aging) and sections (DA) during dry aging process. Sigmoidal trendlines were fit to indicate the change with aging time. StDA: ML = 15.77/{1+exp[‐(day‐3.13)/0.85]}; R^2^ = 0.93; *p* < 0.001. DA: ML = 25.02/{1+exp[‐(day‐15.14)/4.93]}; R^2^ = 0.97; *p* < 0.001.

The average mass loss due to evaporation during conventional dry aging (DA) and steak dry aging (StDA) is shown in Figure 3. As expected, mass loss was faster and more pronounced in the StDA treatment, whereas DA required a longer period to reach the same percentage loss, achieving approximately 15% only after 17 days of storage. This difference is attributed to the thinner StDA samples, in which moisture loss occurs primarily through the exposed cut surface. The drying rate is higher in thinner cuts due to their elevated surface‐to‐volume ratio (Álvarez et al. 2023), which facilitates rapid water migration from the interior to the surface without significant internal resistance, resulting in accelerated evaporation.

Mass losses due to evaporation were significantly greater (*p* < 0.05) in the DA samples (Table 1), with final values like those reported for dry‐aged boneless beef in previous studies (DeGeer et al. 2009; Zhang et al. 2025). In the StDA samples, exudation losses (purge) occurred during the wet aging stage, with significantly greater (*p* < 0.05) liquid accumulation in samples aged for 28 days compared to 14 days, slightly lower than values reported by Guimarães et al. (2024) and Yu et al. (2023) for 28‐day wet‐aged beef. This increase is likely due to greater degradation of myofibrillar and cytoskeletal proteins in these samples (higher MFI values; Table 1), leading to the loss of structural integrity between myofibrils and the cell membrane, which promotes increased water migration to the extracellular space (Aroeira et al. 2017).

**TABLE 1:** Average means of mass losses, retail yield and physicochemical characteristics of beef striploins (M. *L. lumborum*) unaged and aged by different methods.

		Aging treatments				
Characteristics	Unaged	StDA14	StDA28	DA	SEM	Pr > F
Mass loss (%)						
Evaporation	—	14.97*^b^*	16.03*^b^*	23.67*^a^*	0.77	*<0.001*
Exudation (purge)^1^	—	0.78*^x^*	2.31*^y^*	—	0.19	*<0.001*
Trimming (crust)^2^	—	—	—	26.32	0.82	—
Retail yield (%)	—	84.36*^a^*	82.03*^a^*	44.12*^b^*	3.44	*<0.001*
Chemical composition (%)						
Protein	22.03*^c^*	25.04*^b^*	25.60*^ab^*	26.00*^a^*	0.28	*<0.001*
Moisture	70.87*^a^*	66.14*^b^*	65.17*^bc^*	64.60*^c^*	0.41	*<0.001*
Fat	4.00*^c^*	4.19*^bc^*	4.39*^ab^*	4.58*^a^*	0.36	*<0.001*
Ash	3.10*^b^*	4.63*^a^*	4.84*^a^*	4.82*^a^*	0.19	*<0.001*
Water activity, aw	0.986*^a^*	0.981*^b^*	0.981*^b^*	0.979*^b^*	0.001	*<0.001*
Water holding capacity, WHC (%)						
EZ‐DripLoss, EZ‐DL	2.57*^a^*	0.03*^b^*	0.06*^b^*	0.10*^b^*	0.19	*<0.001*
Cooking loss, CL	27.84*^a^*	17.87*^c^*	17.44*^c^*	21.58*^b^*	0.81	*<0.001*
Myofibrillar fragmentation index, MFI	18.84*^d^*	31.65*^c^*	43.70*^b^*	58.18*^a^*	2.63	*<0.001*
WBsSF (N)	63.90*^a^*	35.52*^b^*	35.57*^b^*	27.45*^c^*	2.45	*<0.001*

StDA14 = beef steaks dry‐aged for 6 days, vacuum‐packed and aged for 8 days; StDA28 = beef steaks dry‐aged for 6 days, vacuum‐packed and aged for 22 days; DA = beef sections dry‐aged for 28 days; WBsSF = Warner‐Bratzler square shear force; and SEM = standard error of the mean (*n* = 10 per treatment).

^1^ After vacuum‐packaging aging (purge did not apply to DA treatments).

^2^ Trimming losses were evaluated only on DA samples since they did not apply to StDA treatments.

^x,y^ Means followed by different letters in the row differ (*p* < 0.05) by F test.

^a‐d^ Means followed by different letters in the row differ (*p* < 0.05) by Tukey test.

The trimming losses were not evaluated in the StDA treatments, since the thin crust formed during dry aging was rehydrated during the wet aging phase by exudate migrating from the meat's interior. In contrast, DA samples exhibited mass losses related to crust removal that were comparable to the evaporation losses (Table 1). Such losses in DA are well‐documented (Haddad et al. 2022; Guimarães et al. 2024; Zhang et al. 2025). Therefore, the absence of trimming in StDA samples substantially reduced processing losses, resulting in significantly higher saleable yields—at least 35% greater than those of DA samples (Table 1).

Therefore, the StDA process has proven to be a highly efficient strategy among technological approaches aimed at minimizing losses and improving the profitability of dry aging. Reductions of only 1%–4% in evaporation losses and 2%–3% in trimming losses have been reported for dry aging in water‐permeable bags (Ahnström et al. 2006; Dikeman et al. 2013; Li et al. 2014; Setyabrata et al. 2022). In stepwise aging processes, similar reductions (2%–3%) in evaporation and trimming losses were reported when using water‐permeable bag dry aging (Kim et al. 2017; De Faria Vilella et al. 2019).

### 3.2 Chemical Composition, Water Activity, and Water Holding Capacity

As expected, the moisture content of the samples decreased during dry aging, and values for the DA samples did not differ significantly (*p* > 0.05) from those of the StDA28 samples (Table 1). A similar pattern was observed for protein and fat content, which can be attributed to a concentration effect. The reduction in moisture content leads to a relative increase in protein and fat per unit of total mass, without representing an actual increase in these constituents (Laster et al. 2008; Berger et al. 2018). Likewise, water activity (aw) decreased with aging but was not affected by the treatments (*p* > 0.05). Lower aw values during dry aging result from the reduction in free water due to higher evaporation losses, as reported by other authors (Da Silva Bernardo et al. 2020; Haddad et al. 2022).

Regarding water‐holding capacity (WHC), no significant differences (*p* > 0.05) were observed in drip losses (EZ‐DL) among treatments, but DA samples exhibited higher (*p* < 0.05) cooking losses (CL) than StDA samples (Table 1). Similar to moisture and aw, lower drip loss and CL would be expected with conventional dry aging due to greater water loss during processing (Da Silva Bernardo et al. 2020; Haddad et al. 2022). One hypothesis that would explain the lower CL values is that the surface proteins of the StDA steak may have undergone thermal denaturation at lower temperatures due to prior denaturation during the dry aging phase (followed by rehydration during the wet aging phase), forming a protective barrier that limited water loss during cooking.

### 3.3 Myofibrillar Fragmentation Index and Shear Force

The myofibrillar fragmentation index (MFI) was significantly affected by the treatments (*p* < 0.05), with greater values observed in aged samples due to increased muscle proteolysis (Table 1). In the StDA samples, proteolysis was greater (*p* < 0.05) in steaks wet‐aged for a longer period (StDA28 > StDA14). However, DA samples exhibited higher MFI values than StDA samples, even compared with StDA28. According to Huff‐Lonergan and Lonergan (2005), several biochemical factors, including the activation of protease inhibitors and/or protein oxidation during post‐mortem aging, can influence the degradation of myofibrillar proteins. It is possible that dry aging in individual steaks (StDA) led to greater protein oxidation compared with conventional dry aging (DA), due to the larger surface area exposed to ambient air, which may have limited proteolytic activity. Ha et al. (2019) reported greater MFI values in samples dry‐aged for 35 and 56 days compared with wet‐aged samples, but no differences were observed between 56‐day dry‐aged and wet‐then‐dry‐aged (21‐day wet‐aged followed by 35‐day dry‐aged) ones.

Despite differences in MFI between StDA treatments, no significant differences (*p* > 0.05) were observed in shear force (WBsSF) values between StDA14 and StDA28, indicating that the magnitude of the differences in myofibrillar fragmentation would not be enough to decrease shear force values or it would not be the only factor affecting this variable (Table 1). In contrast, DA samples showed greater MFI values that were reflected in lower WBsSF indicating more tender meat. Although these differences are likely not perceivable by consumers, as the values ​​remain below the Warner‐Bratzler shear force (WBSF) thresholds for “tender” beef of 42 N (equivalent to WBsSF of 52 N) proposed by Destefanis et al. (2008), and of 31 N (equivalent to WBsSF of 35 N) suggested by Shackelford et al. (1991), a sensorial analysis is still necessary to verify whether the treatments are equivalent in tenderness.

### 3.4 pH, Microbial Load and Lipid Oxidation

Compared with unaged samples, pH values increased significantly (*p* < 0.05) in the StDA28 and DA samples (Table 2), which may be associated with the higher degree of proteolysis indicated by elevated MFI values (Table 1). During aging, pH can rise due to the formation of nitrogenous compounds resulting from proteolytic enzyme activity, as well as ionic migration caused by cellular membrane leakage, leading to an increased net protein load (Boakye and Mittal 1993). Both dry aging and stepwise dry/wet aging have been reported to increase pH in beef samples (De Faria Vilella et al. 2019; Zhang et al. 2019; Zhang et al. 2025), although other studies observed no significant changes in pH during aging (Kim et al. 2017; Haddad et al. 2022; Guimarães et al. 2024).

**TABLE 2:** Average means of microbial load (TBC), lipid oxidation (TBARS values), myoglobin redox forms and CIE color indices of beef striploins (M. *L. lumborum*) unaged and aged by different methods.

		Aging treatments				
Characteristics	Unaged	StDA14	StDA28	DA	SEM	Pr > F
pH value	5.56*^c^*	5.52*^c^*	5.70*^b^*	5.90*^a^*	0.03	*<0.001*
Total bacterial count (log CFU/g), TBC	2.27*^b^*	1.55*^c^*	3.14*^a^*	3.00*^a^*	0.12	*<0.001*
TBARS value (mg MDA/kg)	0.14*^b^*	0.25*^a^*	0.26*^a^*	0.28*^a^*	0.01	*<0.001*
Myoglobin redox forms (%)^1^						
Oxymyoglobin, OMb	77.25*^a^*	47.77*^c^*	60.79*^b^*	80.17*^a^*	2.48	*<0.001*
Deoxymyoglobin, DMb	9.64	13.74	11.82	5.30	1.25	*0.081*
Metmyoglobin, MMb	13.11*^c^*	38.49*^a^*	27.39*^b^*	14.49*^c^*	1.87	*<0.001*
Meat Mb Redox^2^						
Oxymyoglobin, OMb_cvs_	94.55*^a^*	47.29*^c^*	73.59*^b^*	91.86*^a^*	4.12	*<0.001*
Deoxymyoglobin, DMb_cvs_	5.38*^b^*	4.91*^b^*	12.61*^a^*	7.94*^b^*	0.92	*0.007*
Metmyoglobin, MMb_cvs_	0.07*^c^*	47.80*^a^*	13.81*^b^*	0.21*^c^*	4.29	*<0.001*
CIE color indices						
Lightness, L*	43.42*^a^*	36.13*^c^*	38.77*^b^*	43.39*^a^*	0.59	*<0.001*
Redness, a*	22.15*^a^*	12.50*^c^*	17.67*^b^*	21.88*^a^*	0.72	*<0.001*
Yellowness, b*	15.43*^a^*	8.79*^c^*	11.89*^b^*	15.23*^a^*	0.52	*<0.001*
Chroma, C*	27.00*^a^*	15.32*^c^*	21.31*^b^*	26.66*^a^*	0.87	*<0.001*
Hue angle, *h* (°)	34.83	34.85	33.93	34.75	0.48	*0.898*

StDA14 = beef steaks dry‐aged for 6 days, vacuum‐packed and aged for 8 days; StDA28 = beef steaks dry‐aged for 6 days, vacuum‐packed and aged for 22 days; DA = beef sections dry‐aged for 28 days; MDA = malondialdehyde; and SEM = standard error of the mean (*n* = 40).

^1^ Krzywicki (1979) mathematical method.

^2^ Bueno, Silva, et al. (2024) computer vision system (CVS) method.

^a‐c^ Means followed by different letters in the row differ (*p* <0.05) by Tukey test.

Total bacterial counts (TBC) were lower (*p* < 0.05) in StDA14 samples than in all other groups, including unaged samples (Table 2), which may be attributed to the initial surface drying of the steaks during the dry‐aging phase. The formation of this thin crust likely reduced favorable conditions for microbial growth, particularly by limiting free surface water, thereby lowering TBC values. It has been reported that microbial counts increase from day 14 to 28 of dry aging and remain stable thereafter, likely due to reduced aw, especially at the surface, where microorganisms penetrate the tissue (Haddad et al. 2022; Guimarães et al. 2024). Bischof et al. (2023) observed that the total microbial count on surface trimmings of dry‐aged meat increased until day 14 and remained constant until day 28 of aging. Hulánková et al. (2018), however, reported a decrease in total microbial counts both on the surface (crust) and in the internal meat after approximately two weeks of storage, which they attributed to surface drying during aging, since moisture content decreased significantly. In dry‐aged meat, the limiting conditions for microbial growth on the surface take longer to be established, allowing time for microbial growth and adaptation to these conditions. In our experiment, however, surface drying occurs much faster, leaving little time for microbial adaptation and growth, which explains the lower microbial counts compared to non‐aged meat. However, prolonging the wet aging phase allowed microbial multiplication, resulting in TBC in StDA28 samples reaching levels like those of DA samples. Moreover, no differences (*p* > 0.05) were observed between StDA28 and DA treatments and all treatments maintained low microbiological counts (<3.5 log CFU/g), indicating excellent microbiological quality. Typically, off‐flavors from spoiled meat are detectable when bacterial counts reach approximately 7 log CFU/g, considered the upper limit for microbiological acceptability (ICMSF 1986).

Lipid oxidation, measured as TBARS, increased significantly (*p* < 0.05) with aging, but no significant differences (*p* > 0.05) were observed among aged samples (Table 2). Increases in TBARS values with dry aging time have been reported previously (Ha et al. 2019; Liu et al. 2024; Zhang et al. 2025). Regarding aging methods, similar results were observed by Correa et al. (2025), who reported no differences in TBARS between wet‐aged, bag dry‐aged, and stepwise (dry/wet or wet/dry) aged grain‐finished beef.

Lipid oxidation, together with post‐mortem proteolysis, dehydration, and microbial activity, plays an important role in developing the distinctive quality and flavor of dry‐aged beef (Zhang et al. 2022). However, increased lipid oxidation can also produce off‐flavors, particularly rancidity. Campo et al. (2006) associated rancid flavor with TBARS values of 2.0 mg MDA/kg using distillation extraction. Considering that distillation yields TBARS values about 1.35 times higher than aqueous extraction (Pikul et al. 1989), the equivalent threshold for aqueous extraction would be approximately 1.5 mg MDA/kg, still about six times higher than the values observed in this study.

### 3.5 Myoglobin Chemical Forms and Instrumental Color

DA samples exhibited greater (*p* > 0.05) oxymyoglobin (OMb) and lower (*p* > 0.05) metmyoglobin (MMb) values after blooming compared with StDA samples (Table 2), demonstrating clear differences between treatments. Despite absolute value differences, the proportions of myoglobin redox forms were similar between the colorimeter and CVS measurements. However, the CVS system likely provides more reliable information, as it records the proportions across the entire meat surface, accounting for sample heterogeneity, whereas the colorimeter assesses only a limited area (Bueno, Silva, et al. 2024).

The MMb, responsible for the brownish discoloration of meat, is considered the main marker of color deterioration (myoglobin oxidation) and perceived freshness loss. Mancini et al. (2022) reported that trained panelists perceived ground beef as slightly dark red to tannish at a surface MMb content of 37%. In this study, only the StDA samples exceeded this threshold, although extending vacuum aging from 14 to 28 days reduced MMb levels below this limit. The higher MMb content in StDA samples may be attributed to surface drying, which increases the concentration of proteins, salts, and iron. The greater availability of free iron, together with reduced water activity, promotes the oxidation of OMb to MMb. Moreover, solute concentration resulting from water removal induces protein denaturation and the formation of a thin surface layer that limits oxygen diffusion into the meat (Cardoso et al. 2019). With a thinner inner OMb layer due to reduced oxygen penetration, the beef steaks appeared markedly darker after drying (Figure 2C; not measured by colorimeter or CVS). During the prolonged wet aging phase, rehydration of the crust likely activated the meat's reducing system, partially converting MMb to DMb; however, StDA28 samples still had (*p* < 0.05) higher MMb proportions than DA samples.

The mechanisms underlying aging‐related color changes and stability are not yet fully elucidated, as prolonged aging can reduce blooming potential and accelerate surface discoloration (Kim et al. 2018). Nevertheless, both myoglobin redox forms (colorimeter and CVS) and color indices were similar in unaged and DA samples. According to Ribeiro et al. (2021), the water loss during dry aging promotes the shrinkage of myofibrils, increasing the space available for light absorption, then decreasing the meat's light scattering power and contributing to the darkening (lower L* value) of color intensity. However, in this study, the L* values of the DA samples did not differ (*p* > 0.05) from the unaged samples. Guimarães et al. (2024) also observed no changes in L* and hue (*h*) during dry aging but reported increases in a*, b*, and chroma (C*) values. Ribeiro et al. (2021) reported that dry‐aged samples exhibited lower L* values than unaged samples. However, those authors aged the samples for longer periods (42 days), which is sufficient for lipid oxidation to occur (an effect that was not observed in the present study). Kim et al. (2022) reported that L* values of the inner meat surface of dry‐aged and dry‐aged bag beef decreased significantly only after 60 and 45 days of aging, respectively. Therefore, differences with our results may arise from variations in environmental conditions during aging or initial muscle characteristics (e.g., meat freshness, breed, feeding practices, aging time, etc.).

Consistent with myoglobin redox data, all color indices of aged samples differed (*p* < 0.05) except for *h* values (Table 2). DA beef displayed a lighter (*p* < 0.05) lean color (greater L* value) and a more intense red (greater C* value) color than StDA samples. The highest a* and C* values observed for the StDA14 samples, followed by StDA28 and then unaged and DA samples, support the observation by Bueno et al. (2024) that MMb accumulation negatively correlates with a* and C*, reducing redness intensity. Mancini et al. (2022) also reported that a* and C* closely correspond to visual color perception and MMb changes.

**FIGURE 4:**
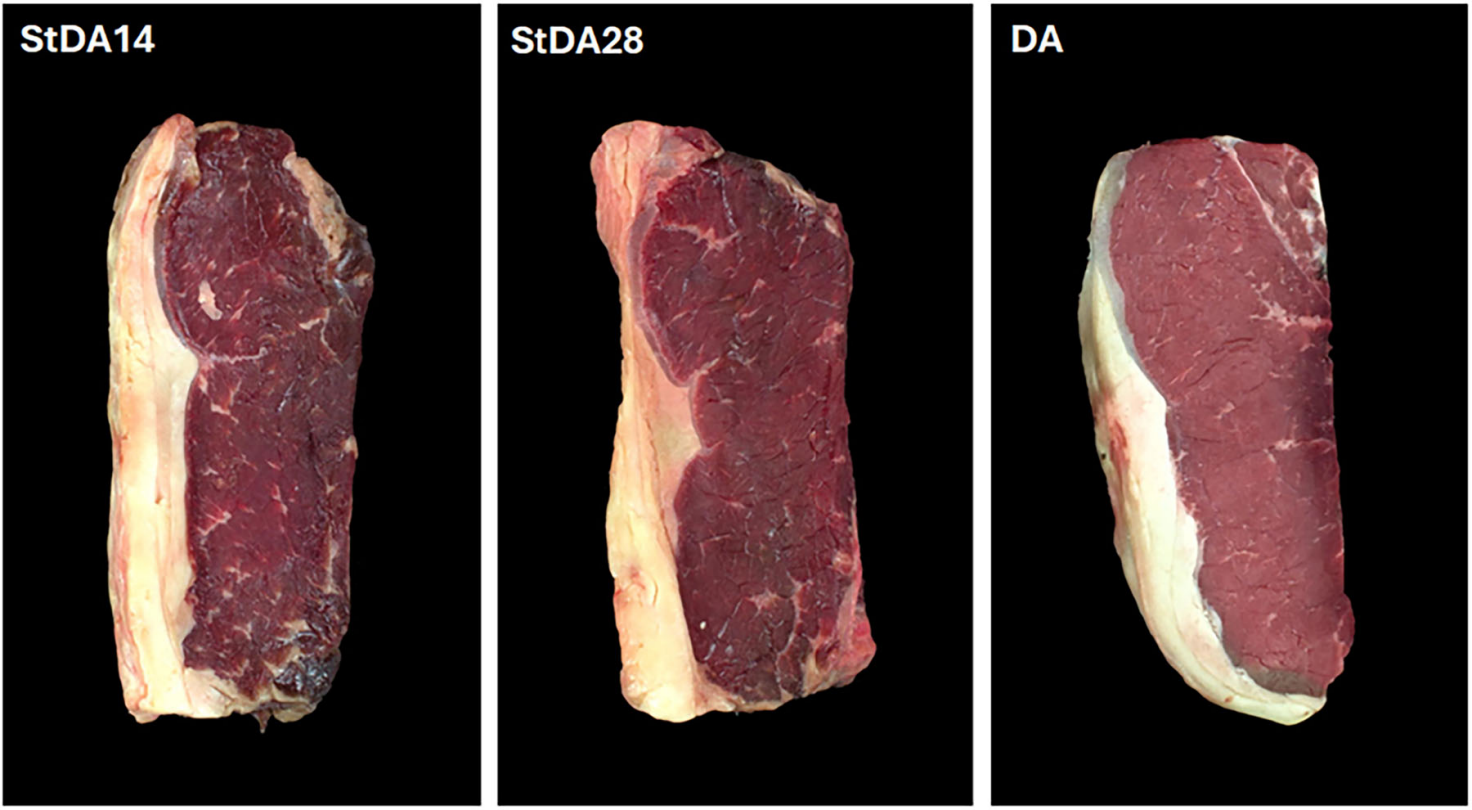
Representative photos of dry‐aged striploin beef steaks by the conventional dry aging (DA) and steak dry aging (StDA) processes. StDA14 = steaks dry‐aged for 6 days, vacuum‐packed and aged for 8 days; StDA28 = steaks dry‐aged for 6 days, vacuum‐packed and aged for 22 days; DA = steak of striploin piece dry‐aged for 28 days.

Samples StDA14 had (*p* < 0.05) a lower L* value than samples StDA28, probably due to less rehydration during the wet aging period. According to Ribeiro et al. (2025), the evaporative moisture loss during dry aging concentrates pigments in the crust, reducing L* and producing a gray–brown surface. Therefore, part of the thin protein layer on the surface remained denatured, concentrating the pigments, reducing light scattering, and promoting MMb formation. Although StDA28 samples appear darker than DA samples (Figure 4), this likely has minimal impact in the Brazilian market, as dry‐aged steaks are typically sold frozen and packaged, showing comparable color indices and virtually no visual difference (Figure S2). Furthermore, Zhang et al. (2019) reported that 12 months of frozen storage minimally affected the quality and acceptability of dry‐aged lean beef, highlighting the commercial viability of StDA steaks. Nevertheless, a consumer study of the acceptance of the appearance and purchase intention of the StDA and DA frozen steaks should be conducted to assess the real market potential.

### 3.6 Free Amino Acids Profile

Table 3 shows the effects of the treatments on the release of free amino acids (FAA). The increase in FAA results from microbial and meat proteolytic activity of aminopeptidases and peptidases during aging, which contributes to muscle tenderization (Terjung et al. 2021). FAA not only directly influences meat flavor but also serves as precursors for aromatic compounds formed during cooking via the Maillard reaction and Strecker degradation (Herrera and Calkins 2022; Lee et al. 2019). Consequently, both the concentration and composition of amino acids provide valuable insights into the observed differences in tenderness and flavor among treatments.

**TABLE 3:** Effects of different methods of aging on the release of individual free amino acid (FAA; mg/g protein) in beef striploins (M. *L. lumborum*) steaks.

		Aging treatments				
Amino acids	Unaged	StDA14	StDA28	DA	SEM	Pr > F
Essential amino acids (EAA)						
Histidine, His	0.53*^c^*	0.69^bc^	0.79*^b^*	1.13^a^	0.07	*<0.001*
Isoleucine, Ile	*nd*	0.44^b^	0.59^ab^	0.73^a^	0.05	*0.040*
Leucine, Leu	0.39^b^	1.06^a^	1.32^a^	1.40^a^	0.13	*<0.001*
Lysine, Lys	*nd*	*nd*	*nd*	0.67	0.09	—
Methionine, Met	*nd*	0.15	0.18	0.21	0.03	*0.169*
Phenylalanine, Phe	*nd*	0.50^b^	0.59^ab^	0.67^a^	0.03	*0.047*
Threonine, Thr	*nd*	0.44^b^	0.60^ab^	0.67^a^	0.05	*0.035*
Valine, Val	*nd*	0.63^b^	0.86^ab^	0.93^a^	0.05	*0.038*
Non‐essential amino acids (NEAA)						
Alanine, Ala	1.58^b^	2.38^a^	2.38^a^	2.26^a^	0.11	*0.005*
Arginine, Arg	14.72	16.55	15.55	13.63	0.44	*0.104*
Aspartic acid, Asp	*nd*	*nd*	*nd*	0.27	0.05	—
Cystine, Cys	*nd*	*nd*	*nd*	*nd*	—	—
Glutamic acid, Glu	*nd*	1.25^ab^	2.30^a^	0.80^b^	0.27	*0.044*
Glycine, Gly	*nd*	0.38^b^	0.46^ab^	0.53^a^	0.02	*0.039*
Proline, Pro	*nd*	*nd*	*nd*	*nd*	—	—
Serine, Ser	*nd*	0.56	0.59	0.60	0.04	*0.930*
Tyrosine, Tyr	*nd*	*nd*	*nd*	*nd*	—	—
Non‐standard amino acids (NSAA)						
Hidroxiproline, Hyp	*nd*	*nd*	*nd*	*nd*	—	—
Taurine, Tau	0.53^b^	0.69^b^	0.59^b^	1.13^a^	0.08	*<0.001*
Σ Free EAA	9.12^b^	14.12^a^	14.01^a^	16.28^a^	0.96	*0.023*
Σ Free NEAA + NSAA	8.38^b^	11.48^a^	12.74^a^	9.32^b^	0.60	*0.010*
Σ Free amino acids	17.50^b^	25.60^a^	26.75^a^	25.60^a^	1.30	*0.011*

StDA14 = beef steaks dry‐aged for 6 days, vacuum‐packed and aged for 8 days; StDA28 = beef steaks dry‐aged for 6 days, vacuum‐packed and aged for 22 days; DA = beef sections dry‐aged for 28 days; *nd =* non‐detected; and SEM = standard error of the mean (*n* = 3 per treatment).

^a‐c^ Means followed by different letters in the row differ (*p* <0.05) by Tukey test.

Although the total FAA content did not differ (*p* > 0.05) between the aged treatments, the DA treatment promoted greater release of histidine (His), isoleucine (Ile), phenylalanine (Phe), threonine (Thr), valine (Val), and glycine (Gly) compared to StDA14. Many of these amino acids were also released in the StDA28 samples, resulting in an FAA profile resembling that of the DA treatment, which corroborates the MFI data (Table 1) and indicates more intense proteolysis in these treatments. While amino acids such as Ile, leucine (Leu), Phe, and Val are often associated with bitter taste, Thr, serine (Ser), methionine (Met), and Gly contribute to sweet taste, which can counterbalance bitterness and provide a more balanced and complex flavor profile (Khan et al. 2015). Therefore, extending the wet aging period from 14 to 28 days was likely important for developing flavor characteristics like those of the DA samples.

No differences (*p* > 0.05) were observed in the sum of essential amino acids (EAA) among aged treatments. However, the StDA samples exhibited greater (*p* < 0.05) concentrations of the sum of non‐essential (NEAA) and non‐standard (NSAA) amino acids than DA treatment. Notably, the StDA28 treatment showed the highest glutamic acid/glutamine (Glu) content, one of the main contributors to umami (meaty) taste, which is directly associated with characteristic meat flavor (Iida et al. 2016). Umami compounds act as important flavor enhancers, and a continuous increase of Glu has been reported up to 50 days of the dry aging process (Lee et al. 2019; Iida et al. 2016). However, aspartic acid/asparagine (Asp), also related to umami and sour tastes (Xu et al. 2023), was detected only in DA samples. The DA samples had the highest release of NSAA taurine (Tau). Guimarães et al. (2025) observed that conventionally dry‐aged samples were associated primarily with the sensory descriptor “toast” taste and “grilled/barbecue” aroma, which in turn were associated with the higher concentration of the amino acids Phe and Tau in the meat exudate.

## 4 Conclusion

The “Steak Dry Aging” (StDA) method represents an effective and innovative alternative to conventional dry aging, promoting significant yield improvements and reducing losses without compromising microbiological, physicochemical, or amino acid profiles when the total aging period is the same (28 days). This approach accelerates initial dehydration and eliminates the need for trimming by rehydrating the surface crust, optimizing the utilization of the dry aging chamber, and enabling shorter production cycles, as the wet aging period can coincide with product distribution and marketing. These findings indicate that StDA is feasible for implementation in small‐ and medium‐sized processing facilities, enhancing cost‐effectiveness, production scalability, and broader access to dry‐aged beef.

Nevertheless, there remains potential to further optimize the technique to improve overall meat quality. Future studies should evaluate factors such as animal breed/genotype and age, as well as process adjustments—particularly relative humidity and airflow control during the dry aging stage—to determine their effects on appearance, sensory attributes, and consumer acceptance.

## Author Contributions

**Jean Carlos dos Santos**: conceptualization, methodology, investigation, visualization, writing – original draft. **Angélica Sousa Guimarães**: methodology, investigation. **Lorrany Ramos do Carmo**: investigation. **Márcia Cristina Teixeira da Silveira**: resources. **Alcinéia de Lemos Souza Ramos**: formal analysis, writing – review and editing. **Leandro Sâmia Lopes**: validation, supervision, writing – review and editing. **Eduardo Mendes Ramos**: conceptualization, methodology, supervision, funding acquisition, project administration, writing – review and editing.

## Conflicts of Interest

The authors declare no conflicts of interest.

## Supporting information




**Supplementary Figure S1‐S2**: jfds71029‐sup‐0001‐FigureS1‐S2.pdf
