# Supplementary material for: “Steak Dry Aging”: An Innovative Approach to Producing High‐Yield, High‐Quality Dry‐Aged Beef
Source: J Food Sci. 2026 Mar 28;91(4):e71029. doi: 10.1111/1750-3841.71029 (PMC13032162; doi:10.1111/1750-3841.71029)
Supplement: Supplementary file 1 — Supplementary Figure S1‐S2: jfds71029‐sup‐0001‐FigureS1‐S2.pdf [file JFDS-91-0-s001.pdf]

SUPPLEMENTARY MATERIAL

"Steak dry aging": An innovative approach to dry aging beef

Santos et al. (2025)
Journal Food Science

Corresponding author:
Eduardo M Ramos (emramos@ufla.br)
Department of Food Science, School of Agricultural Sciences of Lavras, Federal University of Lavras, Lavras, Minas Gerais, 37200-900, Brazil

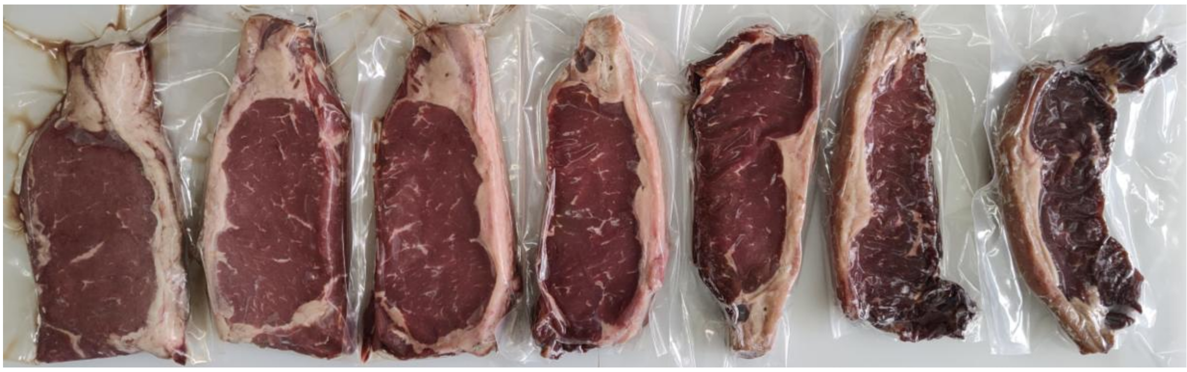

Figure S1. Photos of steaks dry-aged to different degrees of drying, vacuum-packed and wet-aged (at 4 °C) to complete 28 days of total aging.
From left to right: wet-aged sample; and dry-aged with evaporation loss of 6.2%, 9.8%, 18.9%, 21.7%, 25.5% and 31.0% days prior to vacuum-packaging and wet aging period.

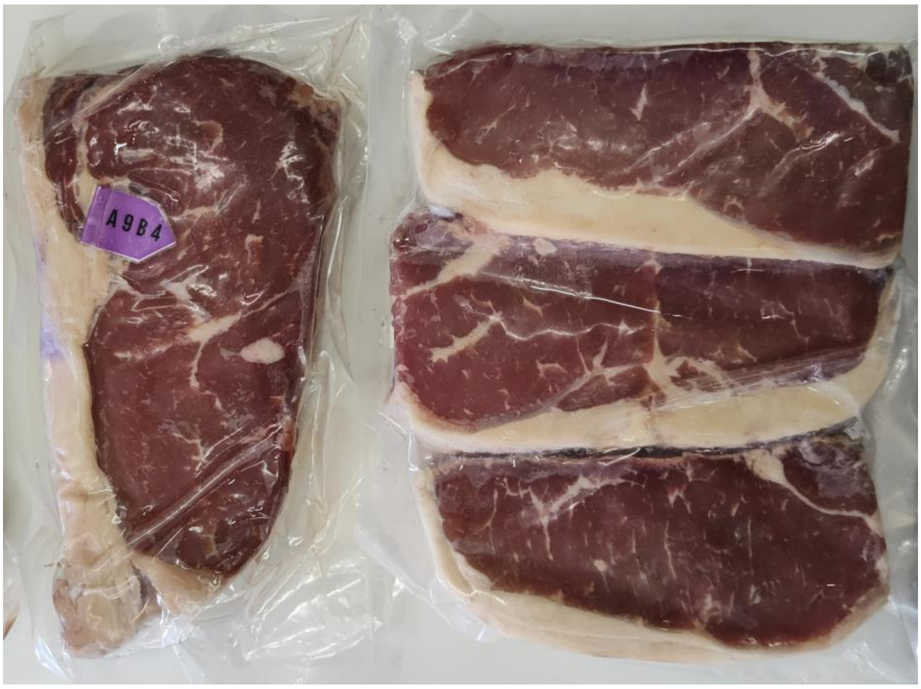

Figure S2. Photos of vacuum-packed steaks aged in the StDA28 (left) and DA (right) processes.

DA = steaks of beef sections dry-aged for 28 days and vacuum-packed; and StDA28 = beef steaks dry-aged for 6 days, vacuum-packed and wet-aged for 22 days.
